# Gestational diabetes alters prefrontal neurochemistry and disrupts maternal behaviors: Role of Fibrillin-1, serotonin, and TNF-α in rats

**DOI:** 10.1016/j.metop.2025.100422

**Published:** 2025-11-17

**Authors:** Samira Khayat, Hamed Fanaei, Abdolvahed Safarzaei

**Affiliations:** a Pregnancy Health Research Center, Zahedan University of Medical Sciences, Zahedan, Iran; b Cellular and Molecular Research Center, Research Institute of Cellular and Molecular Sciences in Infectious Diseases, Zahedan University of Medical Sciences, Zahedan, Iran; c Department of Physiology, School of Medicine, Zahedan University of Medical Sciences, Zahedan, Iran; d Pharmacology Research Center, School of Medicine, Zahedan University of Medical Sciences, Zahedan, Iran

**Keywords:** Gestational diabetes, FBN1 gene, TNF-Alpha, Serotonin, Maternal behavior, Prefrontal cortex

## Abstract

**Objective:**

Gestational diabetes mellitus (GDM) is a metabolic disorder that can impact various aspects of maternal behavior and neurochemical processes. This study aimed to investigate the effects of GDM on FBN1 (fibrillin-1) gene expression, TNF-alpha, serotonin and maternal behavior in rat.

**Materials and methods:**

A total of twenty female Wistar rats were randomly divided into two groups: the control group and the gestational diabetes mellitus (GDM) group. The study compared maternal behavior patterns between the GDM and control groups and measured the following in the hippocampus and prefrontal cortex: TNF-α and serotonin levels (via ELISA), and FBN1 mRNA expression (via qRT-PCR).

**Results:**

The findings demonstrated that TNF-α levels (*P* = 0.0025) were significantly higher in the prefrontal cortex of the GDM group compared to the control group, whereas serotonin levels (*P* = 0.0037) were significantly lower. Additionally, *FBN1* mRNA expression levels (*P* = 0.012) in the prefrontal cortex of GDM group were significantly higher than those in the control group. In terms of maternal behavior, the GDM group exhibited weakened behaviors compared to the control group. Specifically, endurance of maternal behaviors such as the duration of breastfeeding (P = 0.024), nesting (P = 0.016), and pup grooming (P = 0.017) were significantly decreased in the GDM group compared to control group. The speed of integration of maternal behaviors, specifically the latency to onset of pup retrieval (P = 0.0018), significantly increased, while the number of breastfeeding instances (P = 0.0026) significantly decreased in the GDM group compared to the control group. Furthermore, the emotionality (self-calming) aspect of maternal behavior, specifically self-grooming, exhibited a significant decreases in both duration (P = 0.0097) and number of instances (P = 0.0029) in the GDM group compared to the control group. In the hippocampus, only TNF-α levels were significantly elevated in the GDM group (P = 0.0003); no significant differences were found in serotonin or FBN1 mRNA expression.

**Conclusion:**

These results demonstrate that GDM significantly dysregulates prefrontal cortex neurochemistry (increasing TNF-α and FBN1 mRNA while decreasing serotonin) and profoundly weakens maternal behavior, affecting its endurance, speed of integration, and emotional components in rats.

## 1 Introduction

Gestational diabetes mellitus (GDM) is a common metabolic complication of pregnancy, characterized by glucose intolerance with onset or first recognition during gestation. Beyond its well-documented metabolic consequences, there is increasing recognition that GDM also impacts maternal neurobiological processes and behavior, influencing maternal-infant bonding and possibly exerting long-term effects on offspring development [1,2]. GDM alters the maternal metabolic milieu, resulting in chronic low-grade inflammation and dysregulation of several neuroendocrine pathways. Elevated levels of pro-inflammatory cytokines, most notably tumor necrosis factor-alpha (TNF-α), have been consistently reported in both the periphery and central nervous system of GDM subjects. These cytokines not only exacerbate insulin resistance but also have profound effects on neurotransmitter systems such as serotonin, impacting mood, cognition, and behavioral states [3,4]. In the central nervous system, TNF-α upregulates the serotonin transporter (SERT) expression and activity in astrocytes, leading to enhanced serotonin uptake and reduced synaptic availability, which may underlie mood and behavioral disturbances observed in mothers with GDM [4]. Emerging clinical and animal research illustrates that GDM can impair maternal behaviors—such as nursing, grooming, and pup retrieval in rodents, or mother-infant relationship and bonding quality in humans [1,2,5]. The mechanisms underpinning these behavioral deficits likely involve neuroinflammatory processes and neurotransmitter dysregulation.

Peripheral inflammation in gestational diabetes mellitus (GDM) can trigger neuroinflammation through a cascade involving cytokine signaling, microglial activation, and alteration of the blood–brain barrier (BBB) [6,7]. GDM is characterized by elevated maternal levels of proinflammatory cytokines such as TNF-α, IL-1β, and IL-6, often driven by adipose tissue macrophage activation and oxidative stress. These peripheral mediators cross or signal through the BBB, activating endothelial and microglial cells in the maternal and fetal brain, thereby initiating central immune responses. Experimental studies have shown that GDM-induced inflammation upregulates NF-κB and JNK pathways in hypothalamic and hippocampal tissues, leading to chronic gliosis, oxidative stress, and neuronal structural disruption [6]. Additionally, chronic peripheral inflammation alters adipokine balance—reducing anti-inflammatory adiponectin while increasing leptin and resistin—which further contributes to central inflammatory milieu and insulin signaling impairment in neural cells. Collectively, these pathways link systemic metabolic inflammation in GDM to persistent neuroinflammatory and neurodegenerative processes that may underlie cognitive and behavioral effects in both mother and offspring [6,7].

Serotonin, known for its central role in affect, reward, and social behaviors, has been found to be significantly depleted in relevant brain regions during GDM, potentially disrupting maternal caregiving behaviors [3,4]. Recent attention has focused on the fibrillin-1 (FBN1) gene and its hormonal derivative, asprosin, as critical modulators of metabolic and neurobehavioral responses in the context of GDM. FBN1 encodes a structural extracellular matrix glycoprotein involved in tissue development, elasticity, and TGF-β signaling modulation [8]. Genetic and epigenetic regulation of FBN1 is altered in metabolic disorders, including GDM, suggesting a wider functional remit beyond connective tissue integrity [9]. Overexpression of FBN1 has been linked to changes in neuroinflammation, blood-brain barrier function, and stem cell activity in the brain, pointing to potential roles in neurodevelopment and behavior [8,10]. Asprosin, a fasting-induced glucogenic hormone derived from FBN1, has been identified as a key glucose sensor and regulator of appetite. Elevated circulating asprosin levels are a consistent finding in both human and rodent models of GDM [[11], [12], [13], [14]]. Asprosin's central effects extend to activation of appetite-regulating neurons and modulation of hypothalamic-pituitary-adrenal (HPA) axis responses, suggesting possible links to maternal drive, energy allocation, and behavioral adaptation during pregnancy and lactation [13]. High asprosin concentrations positively correlate with increased body weight, food intake, and indices of insulin resistance in pregnant and GDM subjects [12,13]. Experimental data further highlight that asprosin and FBN1 expression can be modulated by metabolic interventions (e.g., metformin) and neuroendocrine factors such as oxytocin, which is itself implicated in maternal behavior regulation [15,16]. The convergence of metabolic signals, inflammatory cytokines, and extracellular matrix proteins like FBN1 produces a complex neurobiological environment in GDM [[17], [18], [19]]. Enhanced TNF-α and altered FBN1/asprosin signaling may culminate in serotonergic and other neurotransmitter disturbances, with downstream consequences for maternal care, emotionality, and bonding behaviors [3,4,17,18]. Observational studies confirm that GDM is associated with reduced maternal-infant relationship quality, elevated risk for perinatal mood disorders, and enduring behavioral problems in the offspring [1,2,20]. Understanding the molecular connections between FBN1 activity, inflammatory mediators (TNF-α), neurotransmitters (serotonin), and maternal behavior is essential for unraveling the neurobiological sequelae of GDM. Such insights may not only inform strategies for the early diagnosis and risk stratification of GDM—using markers such as asprosin—but also guide interventions aimed at supporting maternal mental health and nurturing optimal mother-infant relationships in affected populations [1,12]. Focusing on a critical knowledge gap, this research explores the role of FBN1 in the maternal prefrontal cortex and hippocampus in the context of GDM. We examine how FBN1 interacts with inflammatory and serotoninergic systems and how these interactions mechanistically connect altered metabolic pathways to the spectrum of maternal behaviors, utilizing controlled animal models.

## 2 Materials and methods

For this study, a group of 20 female rats from the white Wistar strain, weighing between 200 and 220 g, were selected. The rats were housed in the animal facility of the Laboratory Animal Research Center, Zahedan University of Medical Sciences, where they were provided with unrestricted access to food and water. The rats were maintained under controlled environmental conditions, including a 12-h light-dark cycle and a temperature of 22 ± 2 °C. The ethical aspects of the study were approved by the Ethics Committee of Zahedan University of Medical Sciences (ethical code: IR.ZAUMS.AEC.1402.019). Rats were selected by simple random method and divided into 2 groups of 10. Two groups were used in this study as follows.

1) Control group: animals in this group experienced the pregnancy process without any intervention.
2) The Gestational Diabetes Mellitus (GDM) group consisted of pregnant rats that had been induced with gestational diabetes.

### 2.1 Induction of gestational diabetes

Prior to mating, rats in the GDM group were feed a high-fat and high-sugar (HFHS) diet for a duration of six weeks [14,21]. After the six-week period, female and male rats were mated to induce pregnancy, with the first day of pregnancy confirmed through vaginal smear examination. [22,23]. Once pregnancy was confirmed, rats in the GDM group underwent an 8-h fasting period, followed by an intraperitoneal injection of 25 mg/kg Streptozotocin [14,21]. Rats with a venous fasting blood glucose (FBS) level exceeding 120 mg/dL on the fourth day of pregnancy were selected as the GDM model [14,21].

### 2.2 Measurement of maternal behavior

The assessment of maternal behavior was conducted on postnatal day (PND) 2, during a fixed 2-h window in the early light phase (9:30 to 11:30 a.m.), to evaluate behavior at its peak intensity while controlling for diurnal variation [24,25]. To eliminate confounding effects of litter size on maternal care, all litters were standardized to eight [8] pups on PND 1. Dams were briefly separated from their pups, and the pups were carefully scattered throughout the home cage, outside the nest [24,25]. The dam was then promptly reintroduced to the cage, and her behavior was video-recorded for a 60-min observation period. All subsequent behavioral scoring was performed by two trained observers who were blind to the experimental group assignment (Control vs. GDM). Inter-rater reliability was established prior to full analysis. Both observers independently scored a randomly selected subset of videos (20 % of the total, balanced across groups). Agreement was quantified using Cohen's Kappa (κ) for categorical events (e.g., onset of retrieval, κ > 0.85) and Intra-class Correlation Coefficients (ICC) for continuous measures (e.g., durations and frequencies, ICC >0.90). After confirming high reliability, the remaining videos were divided between the raters for scoring.

The following maternal behaviors were monitored and documented.

A. **Endurance-related behaviors (measured as total duration in seconds):**
  • **Nest Building:** The total time the dam spent constructing the nest using her mouth and forepaws.
  • **Breastfeeding:** The total time the dam spent in a nursing posture, either crouching over or lying next to the pups, allowing them access to the nipples.
  • **Pup Grooming:** The total time the dam spent licking the pups, including body and anogenital licking.
  • **Frequency of Pup Grooming:** The number of discrete pup-grooming bouts.
B. **Behavioral Integration (measured as frequency and latency):**
  • **Latency to Onset of Pup Retrieval:** The time (in seconds) from the dam's reintroduction into the cage until she picked up and moved the *first* pup. This measures the speed of initiating maternal care.
  • **Duration of Pup Retrieval:** The total time (in seconds) spent actively retrieving *all* pups, measuring the persistence of the behavior once initiated.
  • **Frequency of Nest Building:** The number of discrete nest-building bouts.
  • **Frequency of Breastfeeding:** The number of times the dam assumed a nursing posture.
  • **Frequency of Pup Retrieval:** The number of times the dam retrieved pups.
C. **Self-directed, Emotionality Behaviors (measured as duration and frequency):**
  • **Self-Grooming:** The total duration (in seconds) and the number of bouts the dam spent grooming her own body. This behavior is considered a marker of self-calming and displacement activity.

### 2.3 Tissue collection and micro-dissection

At the conclusion of the study (on PND 2, after behavioral testing), the dams were deeply anesthetized (ketamine (100 mg/kg) and xylazine (10 mg/kg)). They were then transcardially perfused with ice-cold phosphate-buffered saline (PBS) to clear blood from the cerebral vasculature. The whole brain was rapidly extracted from the skull and placed on an ice-cold plate for micro-dissection. Using fine surgical tools, the prefrontal cortex (PFC) and hippocampus were identified and meticulously dissected from brain based on anatomical landmarks [26].

To eliminate the contribution of peripheral cytokines from the cerebral vasculature, animals were transcardially perfused with a 300 ml of ice-cold phosphate-buffered saline (PBS) prior to brain extraction and dissection.

### 2.4 Biochemical analysis

The prefrontal cortex and hippocampal tissues were subsequently homogenized, and the concentrations of TNF-α and serotonin were assessed using ELISA kits specifically designed for these measurements (Zell Bio, Germany).

### 2.5 RNA extraction

Initially, tissue samples were homogenized thoroughly. Subsequently, RNA was isolated utilizing the RNX-plus reagent (Sinacolon, Iran) following the manufacturer's protocol.

### 2.6 Synthesis of complementary DNA (cDNA)

After RNA isolation, the purified RNA underwent reverse transcription using a cDNA synthesis kit to generate complementary DNA strands.

### 2.7 Quantitative real-time PCR (qRT-PCR)

The synthesized cDNA templates were amplified in a thermal cycler (Applied Biosystems) using a SYBR Green-based master mix (ParsToos, Iran). Specific primers (detailed in Table 1) were used for gene amplification. PCR efficiency values were determined using the LinReg PCR software.

**Table 1:** Primers used for the analysis of gene expression in tissues of the prefrontal cortex and hippocampus.

Genes	Primer sequence
FBN1	F: 5′-GCTCCTACAGATGCGAATGC-3′
	R: 5′-CAACGGCTTCACACACTGG-3′
GAPDH	F: 5′- AGT GCC AGC CTC GTC TCA TA-3′
	R: 5′- GAT GGT GAT GGG TTT CCC GT-3′

FBN1: Fibrillin-1; GAPDH: glyceraldehyde-3-phosphate dehydrogenase; F: forward; and R: reverse.

### 2.8 Statistical analysis

Statistical analysis was performed employing GraphPad Prism Ver. 8.2 software. Initially, the Kolmogorov-Smirnov test was employed to assess the normality of the data distribution. The results revealed a normal distribution for all variables in both the control and GDM groups (p > 0.05). To compare the control and GDM groups, independent samples t-tests were employed, with a significance level set at p < 0.05.

## 3 Results

### 3.1 Behavioral analysis

**Fig. 1:**
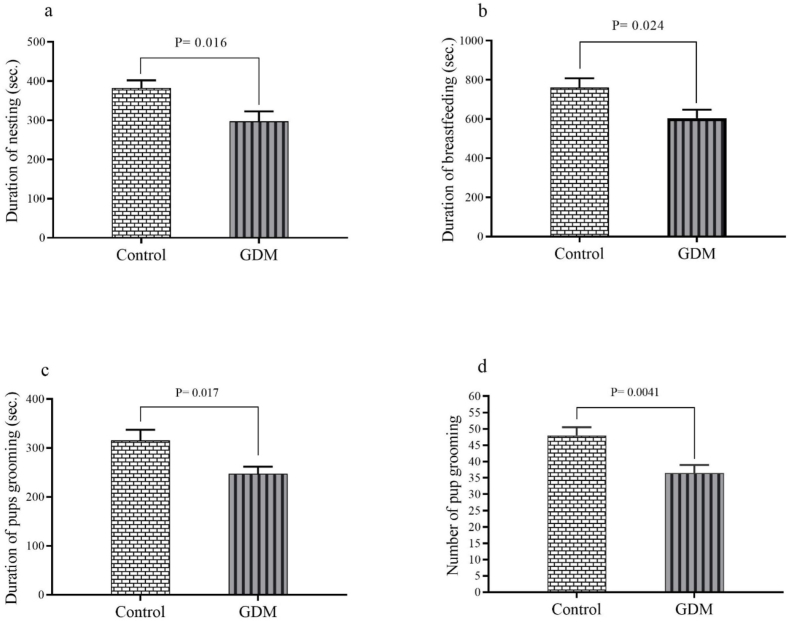
The findings pertain to the endurance of maternal behavior including: (a) duration of nesting, (b) duration of breastfeeding, (c) duration of pups grooming and (d) number of pup grooming. The results are presented as the mean ± SEM.

As shown in Fig. 1A–D, the findings concerning the endurance aspect of maternal behavior revealed a significant decrease in the mean durations of nesting (P = 0.016), breastfeeding (P = 0.024), pup grooming (P = 0.017), and the mean number of pup grooming events (P = 0.0041) in the GDM group compared to the control group.

**Fig. 2:**
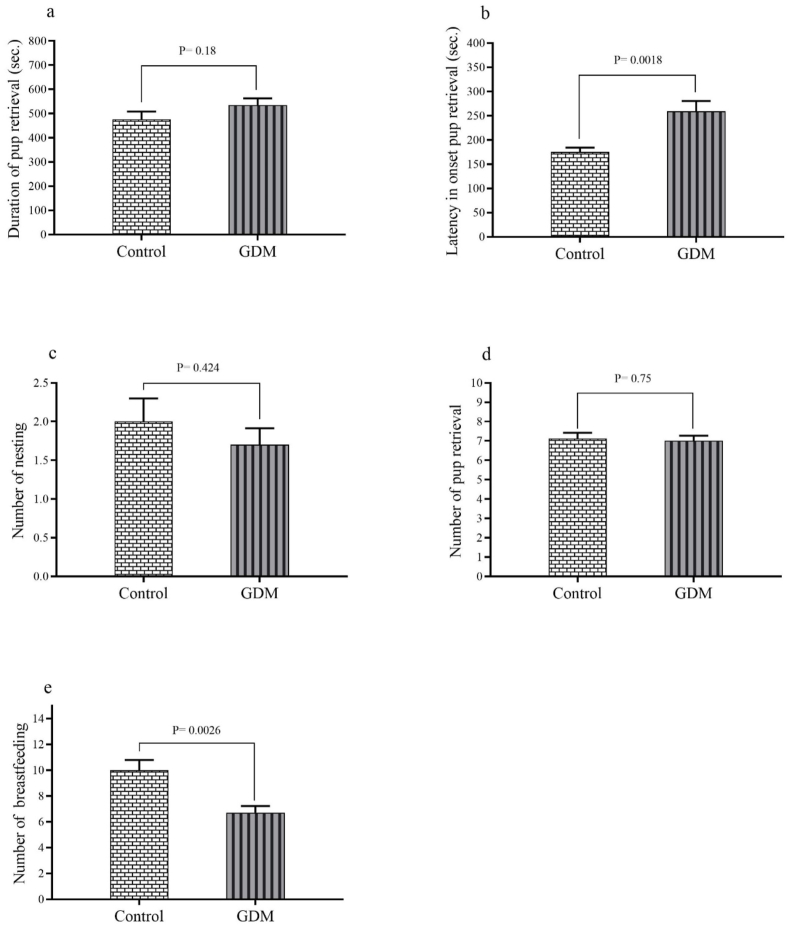
The results relate to the speed of integration of maternal behavior including: (a) duration of pup retrieval, (b) latency in onset pup retrieval, (c) number of nesting, (d) number of pup retrieval, and (e) number of breastfeeding. The results are presented as the mean ± SEM.

As illustrated in Fig. 2a and e, the results pertaining to the speed of integration of maternal behavior exhibited a significant increase in the latency of pup retrieval onset (P = 0.0018) and the significant decrease in number of breastfeeding instances (P = 0.0026) in the GDM group compared to the control group.

**Fig. 3:**
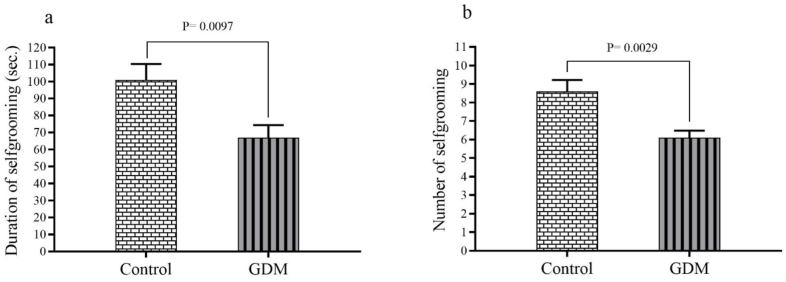
Result relate to emotionality (self-calming or anxiety) of maternal behavior including: (a) duration of self-grooming and (b) number of self-grooming. The results are presented as the mean ± SEM.

Actions related to the emotionality (self-calming or anxiety) (Fig. 3a and b) of maternal behavior indicated a significant decrease in the duration of self-grooming (P = 0.0097) and the number of self-grooming instances (P = 0.0029) in the GDM group compared to the control group.

### 3.2 Biochemical analysis

#### 3.2.1 FBN1 mRNA expression and TNF-α and serotonin concentrations in the prefrontal cortex

**Fig. 4:**
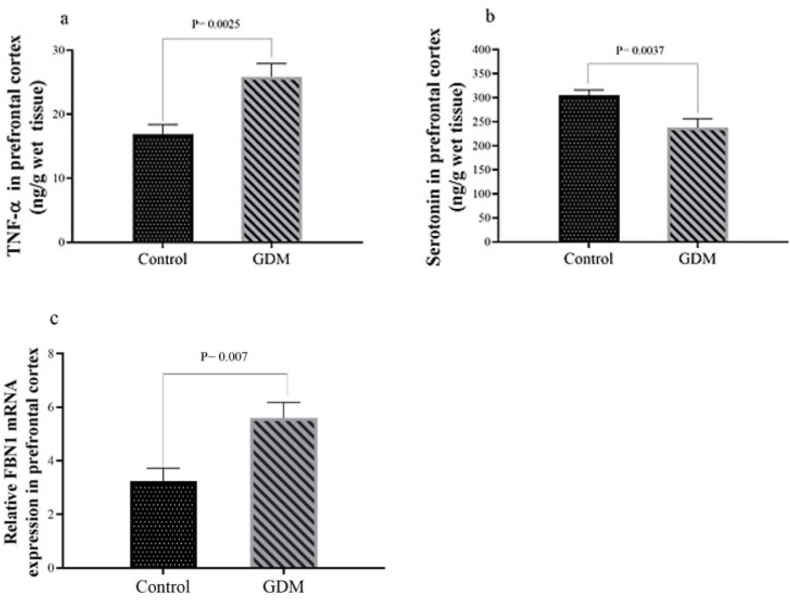
(a) TNF-α concentration, (b) serotonin concentration, and (c) FBN1 mRNA expression in the prefrontal cortex. Results are expressed as mean ± SEM.

The findings in Fig. 4a demonstrate a significant increase TNF-α levels of prefrontal cortex in the GDM group compared to the control group (*P* = 0.0025). Additionally, Fig. 4B shows a significant reduction in prefrontal cortex serotonin concentration in the GDM group relative to the control group (*P* = 0.0037). Furthermore, Fig. 4c reveals a significant increase in prefrontal *FBN1* mRNA expression in the GDM group compared to the control group (*P* = 0.007).

#### 3.2.2 FBN1 mRNA expression and TNF-α and serotonin concentrations in the hippocampus

**Fig. 5:**
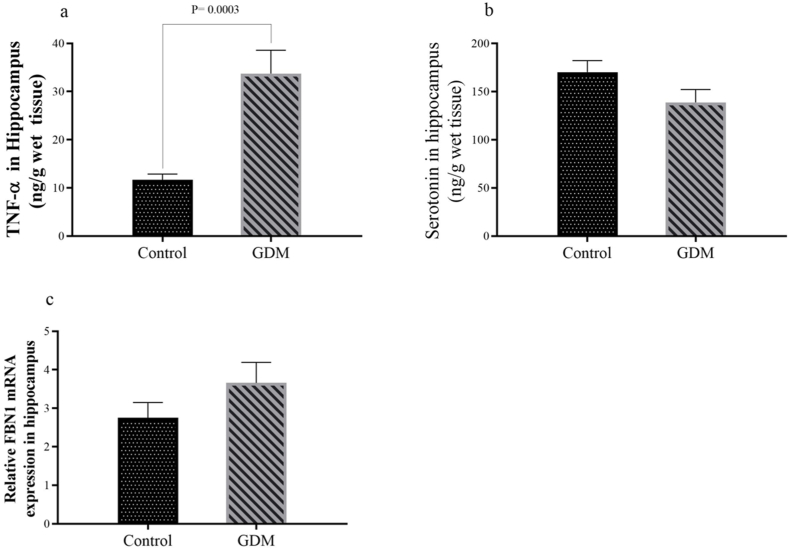
(a) TNF-α concentration, (b) serotonin concentration, and (c) FBN1 mRNA expression in the hippocampus. Results are expressed as mean ± SEM.

A significant increase in hippocampal TNF-α levels was observed in the GDM group compared to controls (Fig. 5a, P = 0.0025). In contrast, reductions in hippocampal serotonin concentration (Fig. 5b, P = 0.0978) and increases in FBN1 mRNA expression (Fig. 5c, P = 0.1929) were also observed in the GDM group, but these changes were not statistically significant.

## 4 Discussion

This study elucidates a novel correlative association between gestational diabetes mellitus (GDM) and maternal behavioral deficits, accompanied by altered FBN1 gene expression, elevated TNF-α, and disrupted serotonin signaling in the prefrontal cortex (PFC).

Our findings underscore the complex neuroimmune and neurochemical interplay that likely underlies impaired maternal caregiving observed in GDM models. Elevated TNF-α levels in the PFC of GDM rats align with accumulating evidence implicating neuroinflammation in the pathophysiology of metabolic and mood disorders. TNF-α, a pro-inflammatory cytokine, can compromise neuroplasticity and neurotransmitter balance through multiple pathways, including modulation of tryptophan metabolism via indoleamine 2,3-dioxygenase activation [27,28]. This link is supported by lower serotonin levels detected here, reflecting either increased degradation or reduced synthesis, consistent with reports of cytokine-induced serotonin depletion in depression and anxiety models [29]. Given the pivotal role of serotonin in maternal behaviors such as nurturing, pup grooming, and anxiety regulation [[24], [30], [31]], these data strengthen the theory that inflammatory cytokines in GDM may disrupt mother-infant bonding and caregiving.

The observed decrease in prefrontal serotonin could result from several inflammatory mechanisms. While we propose the induction of indoleamine 2,3-dioxygenase (IDO) as a potential pathway—shunting tryptophan away from serotonin synthesis and towards the kynurenine pathway—this was not directly tested in the present study and remains a key target for future validation. Alternatively, or concurrently, elevated TNF-α may drive microglial activation, leading to altered synaptic connectivity within serotonergic circuits. Furthermore, GDM-associated inflammation could compromise blood-brain barrier integrity, disrupting the transport of tryptophan or allowing further influx of peripheral inflammatory mediators. The overexpression of FBN1 also suggests a role for extracellular matrix remodeling in this neuroinflammatory milieu, which may modulate cell signaling and contribute to the overall dysregulation of the PFC's neurochemical landscape. Future studies measuring kynurenine pathway metabolites, microglial markers, and BBB integrity are needed to dissect the relative contributions of these mechanisms.

We recognize that the role of FBN1 has been primarily studied in the context of extracellular matrix pathology, and its specific function within maternal behavior circuits remains largely unexplored. Therefore, the investigation of FBN1 in the maternal prefrontal cortex, as presented here, is a novel and exploratory endeavor. Our findings propose a potential link between central FBN1 expression, neuroinflammation, and behavioral deficits in GDM, which now warrants direct future validation.

The overexpression of FBN1 mRNA in the PFC introduces an intriguing dimension to GDM neurobiology. FBN1 encodes fibrillin-1, a large extracellular matrix (ECM) protein serving as a precursor to asprosin, a fasting-induced glucogenic hormone [32,15]. Overexpression of FBN1 may reflect an adaptive or maladaptive response to metabolic stress in GDM, potentially contributing to altered ECM remodeling and neuroinflammation. Supporting evidence indicates that fibrillin-1 deficiency or mutation affects TGF-β signaling and CNS development, which could alter synaptic plasticity critical to maternal behavioral circuits [33]. Conversely, increased FBN1 signaling has been associated with pro-inflammatory microenvironments in metabolic disease [34], suggesting that elevated FBN1 may exacerbate TNF-α-mediated neuroinflammation. These findings resonate with studies showing ECM molecules can modulate neuroimmune responses, affecting behavior [35]. Asprosin, cleaved from FBN1, is emerging as a significant metabolic regulator involved in appetite, stress responses, and glucose homeostasis [[15], [36],^37^]. Elevated asprosin levels have been documented in diabetic and obese individuals, and recent animal models suggest asprosin crosses the blood-brain barrier, acting on hypothalamic neurons to modulate feeding behavior [[15], [36],^37^] [38]. Although direct evidence connecting asprosin to maternal brain function is limited, its known interactions with neuroendocrine axes and energy homeostasis position it as a plausible mediator linking systemic GDM metabolic disruption to altered maternal behavior. Some studies suggest that anti-asprosin therapies may attenuate inflammation and improve behavioral outcomes in metabolic syndrome models [39], warranting future inquiry into its role in gestational neurobiology. Our behavioral data complement the molecular findings, revealing profound deficits in maternal caregiving behaviors such as pup grooming, nursing duration, nesting, and latency to pup retrieval. Reduced self-grooming also points to impaired self-regulatory and emotional coping mechanisms [25], consistent with elevated neuroinflammation and serotonin deficits. Previous rodent studies affirm that elevated TNF-α impairs maternal motivation and pup-directed behaviors [40], while serotoninergic dysregulation exacerbates postpartum anxiety and caregiving deficits [41]. These behavioral disruptions are consistent with the possibility that the altered neurochemical milieu in the PFC associated with GDM contributes to maternal brain circuitry involving oxytocin, dopamine, and serotonin pathways [42,43]. However, FBN1's direct functional role in the adult brain remains underexplored, with most research focusing on its structural role in connective tissue disorders such as Marfan syndrome [44]. Therefore, while our data implicate increased FBN1 expression in the PFC, the precise cellular targets and downstream effects require further elucidation using cell-type specific approaches. Additionally, serotonin alterations in GDM may result from multifactorial influences including tryptophan availability, altered microbiota, or changes in serotonin receptor sensitivity, beyond inflammatory cytokines alone [45,46]. Differentiating between presynaptic versus postsynaptic disturbances could deepen understanding of how serotonin signaling perturbations contribute to maternal behavioral outcomes.

While our data reveal a correlative association between elevated FBN1 mRNA, TNF-α, and decreased serotonin in the PFC, the precise causal relationships remain to be determined. It is plausible that neuroinflammation is a primary driver in this pathway. For instance, elevated TNF-α, a master regulator of inflammation, could directly upregulate FBN1 gene expression as part of a broader reactive response in astrocytes or other glial cells to metabolic stress. Alternatively, the initial metabolic insult of GDM may independently trigger both FBN1 overexpression and neuroinflammation, which then converge to disrupt serotonin signaling. The cleaved product of FBN1, asprosin, has been shown to promote inflammatory signaling in peripheral tissues; if this occurs in the brain, it could create a feed-forward loop where FBN1-derived signals exacerbate TNF-α production, further amplifying serotonin depletion. Future studies utilizing cytokine blockade, FBN1 knockdown, or selective serotonin modulation are necessary to dissect this temporal and mechanistic hierarchy.

The lack of significant changes in hippocampal serotonin and FBN1, in contrast to the profound dysregulation seen in the PFC, highlights a region-specific vulnerability. While our sample size provided sufficient power to detect the large effects in the PFC, it may be underpowered to detect subtler effects in the hippocampus, which should be explored in future studies with larger cohorts.

It is important to note that the GDM model used in this study employs a combination of a high-fat high-sugare diet and a low-dose streptozotocin injection. While this approach is widely used to induce a clinical GDM-like state, we acknowledge that both the diet and the pharmacological agent can independently influence neuroinflammatory pathways and behavior. Future studies incorporating additional control groups (e.g., HFHS diet alone, STZ injection alone on a standard diet) would be valuable to disentangle the specific contributions of the diabetic state from the effects of its inducing factors.

Furthermore, an important and unexamined mechanism in our study is the potential role of gut-brain axis signaling. The HFHS diet used to induce GDM is known to cause significant alterations in the gut microbiota (dysbiosis). Emerging evidence suggests that such diet-induced dysbiosis can promote systemic and central inflammation, influence tryptophan metabolism, and affect the production of microbial metabolites that modulate central serotonin levels [[47], [48], [49]]. These pathways represent a plausible parallel or contributing mechanism to the neuroinflammation and serotonin depletion we observed in the PFC. The interplay between gut microbiota, FBN1/asprosin signaling, and neuroimmune function in the context of GDM is a area for future investigation. Studies profiling the gut microbiome and measuring relevant microbial metabolites in GDM models could provide critical insights into this gut-brain communication pathway and its contribution to maternal behavioral deficits.

## 5 Limitations, translational potential, and practical implications

While this study provides novel insights into the neurochemical and genetic alterations underlying maternal behavioral deficits in gestational diabetes mellitus (GDM), several limitations should be acknowledged. First, the use of a rat model, although valuable for controlled mechanistic studies, may not fully capture the complexity of GDM in humans, including the genetic, environmental, and psychosocial factors that influence maternal behavior. Second, our study examined molecular changes and behavior; future studies using targeted antagonism (e.g., of TNF-α) or genetic knockdown of *FBN1* are necessary to establish direct causality. Third, we focused on a specific brain region and a select set of biomarkers; a broader neurochemical and neuroanatomical investigation could reveal additional pathways involved. Fourth, although this study identified a significant upregulation of FBN1 mRNA in the prefrontal cortex of GDM rats, a key limitation is the absence of direct asprosin measurement. Although FBN1 is the precursor protein for asprosin, future studies quantifying circulating or central asprosin levels are needed to confirm its active role in the observed neurochemical and behavioral disruptions.

Fifth, while our GDM model was based on a well-validated protocol and hyperglycemia was confirmed at the time of model induction, we did not perform longitudinal profiling of peripheral metabolic parameters such as plasma insulin, glucose tolerance, or asprosin levels. Therefore, we cannot definitively rule out that the prefrontal neurochemical changes are solely due to persistent hyperglycemia versus other metabolic factors, nor can we directly correlate peripheral asprosin with central FBN1 expression. Future studies should include comprehensive metabolic phenotyping to better define the relationship between the peripheral diabetic state and its central nervous system sequelae.

Although this study identified a significant upregulation of FBN1 mRNA in the prefrontal cortex of GDM rats, a key limitation is the absence of direct asprosin measurement. Although FBN1 is the precursor protein for asprosin, future studies quantifying circulating or central asprosin levels are needed to confirm its active role in the observed neurochemical and behavioral disruptions. *Furthermore, although we report increased FBN1 mRNA expression, we did not measure asprosin protein levels in either the plasma or the brain. Therefore, the precise role of asprosin in mediating the observed neurochemical and behavioral effects remains hypothetical and requires direct measurement in future studies.*

Despite these limitations, the translational potential of this work is significant. The demonstration that GDM induces neuroinflammatory and neurochemical disruptions that affect maternal behaviors in rats suggests similar mechanisms may operate in humans, given the conserved roles of TNF-α, serotonin, asprosin, and *FBN1* in brain function. Future clinical research should investigate whether similar alterations are present in the plasma or cerebrospinal fluid of women with GDM and whether they correlate with self-reported or observed postpartum depression, anxiety, or bonding difficulties. This could lead to the development of predictive diagnostic tools to identify at-risk mothers during pregnancy.

The practical implications are twofold. From a therapeutic standpoint, our mechanistic data suggest that anti-inflammatory interventions or serotonin-system modulators (e.g., SSRIs) could be particularly relevant for mitigating maternal behavioral deficits in GDM, though this requires extensive clinical validation. More immediately, these findings underscore the critical need for enhanced postpartum support for women with GDM. They strengthen the argument for integrating mental health screening into standard postpartum care for these women, ensuring that disruptions in maternal behavior and bonding are identified early and addressed with psychological support, parenting coaching, and, if necessary, pharmacological treatment. Ultimately, this research highlights that GDM is not merely a peripheral metabolic disorder but a whole-body condition with significant central nervous system consequences, which advocates for a more holistic approach to patient care.

Our behavioral data complement the molecular findings.

The maternal behavioral deficits observed in our GDM rat model find a compelling parallel in recent clinical studies on human mother-infant dyads. A growing body of evidence indicates that GDM in humans is associated with impairments in mother-infant bonding and interaction quality. For instance, clinical studies have reported that mothers with GDM exhibit less affective involvement and reduced bonding [50], while their infants show altered social engagement behaviors [1]. Furthermore, these interaction difficulties can have lasting effects, influencing child neurodevelopment and the mother-child relationship postpartum [51]. Our findings of disrupted pup-directed care (e.g., reduced grooming and nursing) and maternal self-regulation (e.g., reduced self-grooming) in GDM rats provide a potential neurobiological substrate for these clinical observations. The identified dysregulation of prefrontal neurochemistry—specifically the triad of increased FBN1/TNF-α and decreased serotonin—offers a mechanistic hypothesis for the altered maternal motivation and emotional processing observed in clinical settings. However, it is crucial to acknowledge the inherent limitations of the rodent model, which cannot capture the full complexity of human maternal experience, including psychosocial stressors, cultural factors, and the subjective aspects of bonding that are integral to the human condition. Future translational research should aim to correlate these peripheral and central biomarkers with observed and self-reported bonding measures in clinical populations to bridge this gap.

## 6 Conclusion

This study provides novel evidence that gestational diabetes mellitus induces significant neurobiological alterations in the maternal prefrontal cortex, characterized by overexpression of the FBN1 gene, elevated TNF-α levels, and decreased serotonin concentrations in the prefrontal cortex. These molecular changes are closely associated with pronounced impairments in maternal behaviors, including decreased pup grooming, nursing duration, nesting activities, and delayed pup retrieval, alongside diminished maternal self-regulation. The findings suggest that dysregulated FBN1/asprosin signaling is associated with neuroinflammatory pathways and serotoninergic dysfunction which coincide with disruptions in maternal care and bonding mechanisms during GDM. These insights advance our understanding of the neurochemical and genetic basis of maternal behavior deficits in GDM and highlight potential therapeutic targets to improve maternal neuropsychological outcomes and offspring development. Future studies exploring the precise cellular mechanisms and longitudinal impacts of these alterations will be crucial to developing effective interventions for mothers affected by GDM.

While our data position the FBN1/asprosin pathway as a potential therapeutic target, the feasibility and safety of modulating this axis during pregnancy require careful consideration. Preclinical studies in non-pregnant metabolic syndrome models have shown promise; for instance, the use of anti-asprosin monoclonal antibodies has been demonstrated to improve glucose homeostasis and reduce inflammation [39]. However, the translational application of such biologic agents in gestation is complex. Asprosin plays a role in fetal development and metabolic adaptation, and its complete inhibition during pregnancy could have unforeseen consequences on placental function and fetal growth. Therefore, while our findings highlight a novel mechanistic pathway, any future development of interventions targeting FBN1 or asprosin for maternal neurobehavioral benefits would necessitate extensive teratogenicity and safety studies in gestational models. A more immediate and safer translational approach may involve investigating whether lifestyle or dietary interventions that ameliorate GDM also normalize FBN1 expression and its associated neuroinflammatory cascade.

## CRediT authorship contribution statement

**Samira Khayat:** Writing – original draft, Methodology, Investigation, Funding acquisition, Conceptualization. **Hamed Fanaei:** Writing – review & editing, Writing – original draft, Visualization, Supervision, Software, Resources, Project administration, Methodology, Investigation, Formal analysis, Conceptualization. **Abdolvahed Safarzaei:** Methodology, Investigation.

## Ethics approval statement

This study was approved by Ethics Committee of Zahedan University of Medical Sciences (ethical code: IR.ZAUMS.AEC.1402.019).

## Funding sources

Financial support for the study was conducted by the Office of Vice-Chancellor for Research and Information Technology of Zahedan University of Medical Sciences (Grant No. 11119).

## Conflict of interest

All authors declare that they have no actual or potential conflict of interest.

## Data Availability

The data used to support the findings of this study are available from the corresponding author upon request.
